# Insurance Approval for Definitive Proton Therapy for Prostate Cancer

**DOI:** 10.14338/IJPT-21-00002.1

**Published:** 2021-07-27

**Authors:** William M. Mendenhall, Eric D. Brooks, Stephanie Smith, Christopher G. Morris, Curtis B. Bryant, Randal H. Henderson, Romaine C. Nichols, Kathy McIntyre, Stuart L. Klein, Nancy P. Mendenhall

**Affiliations:** 1 Department of Radiation Oncology, University of Florida College of Medicine, Gainesville, FL, USA; 2 University of Florida Health Proton Therapy Institute, Jacksonville, FL, USA

**Keywords:** insurance, proton therapy, prostate cancer

## Abstract

**Purpose:**

To determine factors that influence insurance approval for definitive proton therapy (PT) for prostate cancer.

**Materials and Methods:**

Between 2014 and 2018, 1592 insured patients with localized prostate cancer were evaluated and recommended to undergo definitive PT; 547 patients (34.4%) had commercial insurance, whereas 1045 patients (65.6%) had Medicare/Medicaid. Of those with Medicare, 164 patients (15.7%) had Medicare alone; 677 (64.8%) had supplemental plans; and 204 (19.5%) had secondary commercial insurance. Insurance that “covered” PT for prostate cancer implied that it was an indication designated in the coverage policy. “Not covered” means that the insurance policy did not list prostate cancer as an indication for PT. Of all 1592 patients, 1263 (79.3%) belonged to plans that covered PT per policy. However, approval for PT was still required via medical review for 619 patients (38.9%), comparative dosimetry for 56 patients (3.5%), peer-to-peer discussion for 234 patients (14.7%), and administrative law judge hearings for 3 patients (<0.1%). Multivariate analyses of factors affecting approval were conducted, including risk group (low/intermediate versus high), insurance type (commercial versus Medicare/Medicaid), whether PT was included as a covered benefit under the plan (covered versus not covered), and time period (2014-16 versus 2017 versus 2018).

**Results:**

On multivariate analysis, factors affecting PT approval for prostate treatment included coverage of PT per policy (97.1% had approval with insurance that covered PT versus 48.6% whose insurance did not cover PT; *P* < .001); insurance type (32.5% had approval with commercial insurance versus 97.4% with Medicare; *P* < .001); and time, with 877/987 patients (88.9%) approved between 2014 and 2016, 255/312 patients (81.7%) approved during 2017, and 255/293 patients (87.0%) approved thereafter (*P* = .02). Clinical factors, including risk group, had no bearing on insurance approval (*P* = .44).

**Conclusion:**

Proton insurance approval for prostate cancer has decreased, is most influenced by the type of insurance a patient belongs to, and is unrelated to clinical factors (risk group) in this study. More work is needed to help navigate appropriate access to care and to assist patients seeking definitive PT for prostate cancer treatment.

## Introduction

Definitive proton therapy (PT) is an excellent treatment option for patients with localized prostate cancer. Moreover, PT produces cure rates comparable to both prostatectomy and intensity-modulated radiotherapy (IMRT) but with increasing evidence for reducing late morbidity [1–3]. However, access to PT is limited across the United States with only 30 to 40 proton therapy centers currently in operation.

In addition, access to PT and its benefits is further affected by it being more costly than IMRT and other traditional modalities. This is, in part, due to the increased time and resources needed to effectuate advanced PT technology for a given patient [4–6]. The increased cost with PT can also create potentially vexing discord with insurance coverage, where commercial payors, including nonprofit entities, paradoxically deny PT, despite receipt of treatment comparison plans or other factors demonstrating definitive PT superiority—often at, and despite, the payor's request [7–9]. Similarly, curative intent and other clinical factors supporting PT benefit and indicating medical necessity are also found, paradoxically, to be associated with insurance denial [7–9]. This discrepancy between PT indication, support, and demonstration of superiority to payors, and insurance denial, has drawn questions about the purpose of some of those insurance practices, including prior authorization (PA) and other restrictive payor policies [7–9]. In fact, up to 2 in 3 patients—the majority—will achieve PT approval, but only after appealing and proceeding through the highly restrictive and lengthy insurance denial-overturn process, which differs, depending on the payor [7]. This burden and barrier to care has led some PT centers to strategically redesign operations and devote substantial resources to addressing PA to ensure timely access to appropriate care [10]. During PA or other appeals processes, however, patients are put in positions in which delays in approval may lead to compromised outcomes, including those patients facing prostate cancer.

Although prostate cancer is more indolent than many other malignancies, such as breast and head and neck cancers, delays in treatment may cause anxiety [11]. Additionally, patients with high-risk prostate cancer may feel uncomfortable with the assurance that their malignancy is unlikely to progress through 2 months of androgen-deprivation therapy. Unnecessary delays in care are also against best clinical practice, and there will invariably be patients who achieve worse outcomes because of delays. As such, the denials and the processes to overturn them when PT is indicated affect patients and their care.

Given that many PT centers treat patients with prostate cancer, and the few reports on PT approval for prostate cancer, this study aimed to identify factors affecting the likelihood of insurance approval for definitive prostate PT at the University of Florida Health Proton Therapy Institute (UFHPTI).

## Materials and Methods

Between 2014 and 2018, 1592 insured patients with localized prostate cancer were evaluated at UFHPTI and were deemed to be good candidates for PT. An additional 17 commercially insured patients were excluded from consideration in this series because their insurance company was not a major plan and provided minimal benefits. Patient selection was based on the extent of disease and medical co-morbidities, such that PT would likely reduce the risk of late complications, including radiation proctitis and radiation cystitis and possibly improve the probability of cure [1].

Patients provided consent to participate in an institutional review board–approved outcomes tracking protocol. Additionally, some patients were offered the opportunity to participate in clinical trials, such as a recently initiated Patient Centered Outcomes Research Institute pragmatic trial comparing PT with IMRT (NCT 03561220 [12]), including an embedded optional randomization between 78 Gy in 39 fractions and 60 Gy in 20 fractions for those receiving PT, and a randomized trial comparing PT and IMRT (NCT 01617161 [13]). Patients thought to be at significant risk for pelvic lymph node metastases (approximately 15%–20% or greater) were advised to receive elective pelvic radiation, followed by a boost to the prostate and proximal seminal vesicles [14]. The likelihood of pelvic lymph node metastases was based on the Memorial Sloan Kettering Cancer Center nomogram and the Johns Hopkins Partin tables [15, 16]. Adjuvant androgen-deprivation therapy was often recommended for medically fit patients with high-risk prostate cancer as well as for some with unfavorable intermediate risk disease [17]. Patient characteristics are depicted in **Table 1**.

**Table 1.** Patient characteristics.

**Variable**	**Total, no. (%), N = 1592**	**Commercial plan, no. (%), n = 547**	**Medicare, no. (%), n = 1045**
Risk			
Low	318 (20.0)	133 (24.3)	185 (17.7)
Intermediate	865 (54.3)	305 (55.8)	560 (53.6)
High	409 (25.7)	109 (19.9)	300 (28.7)
T stage			
T1	998 (62.7)	351 (64.2)	647 (61.9)
T2	483 (30.3)	163 (29.8)	320 (30.6)
T3	107 (6.7)	33 (6.0)	74 (7.1)
T4	3 (0.2)	0 (0.0)	3 (0.3)
No data	1 (0.1)	0 (0.0)	1 (0.1)
N stage			
N0	1571 (98.7)	539 (98.5)	1032 (98.8)
N1	21 (1.3)	8 (1.5)	13 (1.2)
M stage			
M0	1588 (99.7)	546 (99.8)	1042 (99.7)
M1	4 (0.3)	1 (0.2)	3 (0.3)
Nodes treated?			
Yes	150 (9.4)	35 (6.4)	115 (11.0)
No	1038 (65.2)	306 (55.9)	732 (70.0)
No data	404 (25.4)	206 (37.7)	198 (18.9)
Androgen-deprivation therapy?			
Yes	306 (19.2)	73 (13.3)	233 (22.3)
No	887 (55.7)	270 (49.4)	617 (59.0)
No data	399 (25.1)	204 (37.3)	195 (18.7)

Insurance status included 547 patients (34.4%) with commercial insurance and 1045 patients (65.6%) with Medicare (including Medicare Advantage). Medicare patients were further stratified according to secondary non-Medicare plans: 164 patients (15.7%) had Medicare alone, 677 patients (64.8%) had supplemental plans, and 204 patients (19.5%) had secondary commercial insurance. The purpose for this breakdown resulted from the recognized differences in processes and approval strategies required to address each form of coverage: supplemental plans, such as AARP and ColonialPenn, are purchased by the patient to provide the 20% co-insurance that Medicare does not pay. Commercial secondary plans, such as BlueCross BlueShield, follow variable rules; some may have a deductible and/or out-of-pocket cost because of prior payments and some may not pay for certain procedures at all, based on plan benefit-coverage stipulations. Notably, many commercial secondary plans are acquired through previous employers after a person has retired, which is common given the average age of prostate cancer diagnoses. Traditionally, supplemental plans tend to cover and approve PT if covered and approved by Medicare, whereas commercial plans have their own independent coverage policies entirely.

Overall, 1263 (79.3%) patients had insurance plans stating coverage for PT, meaning the prostate cancer was a listed diagnosis for which PT could be approved. Conversely, “not covered” meant that prostate cancer was not designated as a diagnosis for which PT could be considered. For those with insurance plans that covered PT, approval was not guaranteed; a lengthy process of medical review and decision appeals often ensued before a final decision of definitive prostate PT, regardless of the plans stated coverage. The process differed notably among insurers. For commercial payors, typically, if a request for approval was denied, there was a first-level appeal, a second-level appeal, followed by an external, independent review organization review. Comparative dosimetry, if requested, was obtained early in the process and was frequently followed by a peer-to-peer discussion. The latter may have been with a radiation oncologist or a physician without significant expertise in oncology. For Medicare plans, a final appeal may have been to an administrative law judge, which is a legal proceeding usually attended by the patient, a representative from the insurance company, and the patient's radiation oncologist or representative. Some patients with prostate cancer pursued all steps in the PA or appeals process, whereas others did not. The endpoint of this study was insurance approval for reimbursement for PT by insurance type, stated coverage of PT, clinical risk group, and time period.

The software JMP Pro (version 14.0.0) was used for statistical analysis (SAS Institute, Cary, North Carolina). Likelihood-ratio χ^2^ analysis assessed any univariate association between binary approval endpoints and risk group (low and intermediate versus high), insurance type (commercial versus Medicare/Medicaid), insurance coverage (covered versus not covered), and time period (2014-16 versus 2017 versus 2018). Time period was included as a variable because it was considered that, as the number of proton facilities increased, insurance policies might change to reflect cost-containing and resource-allocation measures. Thus, 2014-16 was considered the past and 2017 and 2018 were considered to be more recent. Multiple nominal logistic regressions were then used to assess the simultaneous significance of these factors to affect insurance approval.

## Results

### Approval Steps and Process

The steps required in seeking approval for covered versus not-covered patients are depicted in **Table 2**. Patients whose insurance covered protons had few steps required in an effort to obtain approval (see peer to peer [7.8% versus 61.5%], medical review [31% versus 92.2%], comparison plan submission [2.5% versus 10.7%] in **Table 2**). The number of appeals for the group of 1592 patients were as follows: no appeals, 1424 patients (89.4%); 1 appeal, 149 patients (9.3%); and 2 or more appeals, 19 patients (1.2%).

**Table 2.** Steps required in seeking insurance approval for protons for covered versus uncovered patients.

**Variable**	**Covered, no. (%), n = 1387**	**Not covered, no. (%), n = 205)**	**Total, no. (%), n = 1592)**
Consult required before consideration?			
No	1258 (90.7)	143 (69.8)	1401 (88.0)
Yes	129 (9.3)	62 (30.2)	191 (12.0)
Peer-to-peer conducted?			
No	1279 (92.2)	79 (38.5)	1358 (85.3)
Yes	108 (7.8)	126 (61.5)	234 (14.7)
Expedited review?			
No	1323 (95.4)	95 (46.3)	1418 (89.1)
Yes	64 (4.6)	110 (53.7)	174 (10.9)
Medical review?			
No	957 (69.0)	16 (7.8)	973 (61.1)
Yes	430 (31.0)	189 (92.2)	619 (38.9)
Comparison plan submitted?			
No	1353 (97.5)	183 (89.3)	1536 (96.5)
Yes	34 (2.5)	22 (10.7)	56 (3.5)
Court hearing?			
No	1385 (99.9)	204 (99.5)	1589 (99.8)
Yes	2 (<1.0)	1 (<1.0)	3 (<1.0)

### Approval Outcomes by Variable

Overall, 1263 patients (79.3%) had insurance that covered PT. Of those 1263 covered patients, 1227 (97.1%) were ultimately approved; 939 of the 1227 patients (76.5%) whose insurance covered PT and who were approved had a Medicare/Medicaid HMO product. Of the 329 patients whose insurance did not cover PT, approval was obtained for 160 patients (48.6%). Insurance approval for PT by insurance type (commercial versus Medicare) and coverage (yes versus no) is shown in **Table 3**. Approval was obtained for 877/987 patients (88.9%) treated between 2014 and 2016, 255/312 patients (81.7%) treated during 2017, and 255/293 patients (87.0%) treated during 2018.

**Table 3.** Insurance approval for treatment with proton therapy stratified by insurance type and coverage.

**Insurance type**	**Covered (no.)**	**Approved for proton therapy, no. (%)**	**Not approved for proton therapy, no. (%)**
Commercial	Yes (317)	288 (90.9)	29 (9.1)
	No (230)	81 (35.2)	149 (64.8)
Medicare	Yes (946)	939 (99.3)	7 (0.7)
	No (99)	79 (79.8)	20 (20.2)

Univariate analysis approval varied by risk group, *P* = .35; insurance type, *P* < .001; coverage (yes versus no), *P* < .001; and time period, *P* = .01. However, multiple nominal logistic regressions then assessed which of those factors could significantly affect insurance approval when accounting for all other factors. Multiple logistic regressions found approval varied significantly by insurance type, *P* < .001; coverage, *P* < .001; and time period, *P* = .02. Risk group as a clinical correlate had no effect on approval (*P* = .44) (**Table 4**).

**Table 4.** Multiple nominal logistic regressions of variables associated with proton therapy approval.

**Variable**	**Reference level**	**Comparative level**	**Odds Ratio^a^**	**Lower 95%**	**Upper 95%**	***P*** **value**
Risk group	Low/intermediate	High	1.2	0.8	1.9	.44
Time period	2014-16	2017	2.0	1.2	3.3	.02
	2014-16	2018	1.4	0.9	2.4	
Insurance type	Medicare	Commercial	9.9	6.1	15.8	< .001
Protons covered according to policy	Yes	No	21.5	14.1	32.7	< .001

a An odds ratio of >1.0 indicates that the reference level has a higher rate of approval than the comparative level.

After the approval process, treatment was eventually delivered as follows: 1166 patients (73.2%), UFHPTI protons; 21 patients (1.3%), UFHPTI photons; and 405 patients (25.4%), treatment elsewhere or no data.

## Discussion

There are limited data pertaining to insurance approval for PT [7–9]. Even fewer data exist for patients with prostate cancer. Here, we found that approval rates were generally high for patients with prostate cancer at the center: >80%. However, the greatest factor affecting approval was payor type, with Medicare approving significantly more patients than commercial payors. However, up to 1 of 2 patients was able to achieve approval, despite a lack of coverage upfront (48.6%). This highlights findings from other reports in which most noncovered or denied patients are able to achieve approval, but only after a lengthy appeals process and brings attention to how PA and other restrictive policies might act as barriers to care [7–9]. Currently, the process of obtaining insurance approval can be long and arduous and an unknown proportion of patients drop out during the process, settling for alternative therapies rather than risking disease progression during the process. For Medicare, that process can include an administrative law judge, whereas, for commercial and secondary plans, the process can include PA or other approval steps, which can also result in increased time and effort for both centers and patients aiming to achieve appropriate and timely access to care.

We additionally found that, despite reports suggesting or showing PT benefits for prostate cancer [3, 18], PT approval has decreased. This runs in tandem with our finding that PT approval for prostate cancer is not associated with clinical factors, which included risk group at our center. These findings highlight what may be considered overly restrictive payor policies and that the greatest factor influencing approval was insurance type, rather than clinical aspects of care.

Future studies on factors affecting PT access for prostate cancer should be conducted. It is likely that approval varies geographically depending on the health care system and the available medical resources of the country. Furthermore, the health insurance companies in the United States have a financial model that aims to contain costs [19]. The approval of PT for uncommon malignancies, such as pediatric malignancies and liver carcinomas, may be more common, with fewer approvals observed for treatment of more common malignancies, such as breast and prostate cancers. It has been shown in previous studies that the type of payor determines PT approval, rather than clinical factors [7–9], as we observed here. Adding to the approval obstacles encountered by patients is the dilemma that PT remains a scarce resource. In spite of guidelines from the National Comprehensive Cancer Network and others endorsing PT, there remains dissonance in the consensus of whether PT is indicated for specific disease sites or scenarios by stakeholders. It remains to be studied whether part of this disconnect stems from access to PT by stakeholders. Additional challenges remain, and there are differing views on strategies to improve PT access for patients. For example, a potential solution proposed is to address access through index pricing, wherein reimbursement is the same for PT as it is for IMRT. However, the problems with index pricing are that reimbursement for IMRT varies widely, as does the financing of PT facilities, making index pricing fair and feasible for some facilities and not for others. Overall, strategies to successfully navigate and address PT access are ongoing.

One of the limitations of our study is the lack of detailed data related to the time required to secure final insurance approval for each patient. Another limitation is that we do not have data related to participation in a clinical trial and its effect on insurance approval. Finally, we are unsure what proportion of patients who sought approval for PT but did not receive it dropped out in the process because of frustration and fear of disease progression, rather than preference for another therapy.

In conclusion, most patients with prostate cancer have insurance plans that approve PT for the treatment of localized prostate cancer. Factors that affect the likelihood of insurance approval include the type of insurance, whether the policy covers PT, and the time period.
